# Supplementary material for: LinkedSV for detection of mosaic structural variants from linked-read exome and genome sequencing data
Source: Nat Commun. 2019 Dec 6;10:5585. doi: 10.1038/s41467-019-13397-7 (PMC6898185; doi:10.1038/s41467-019-13397-7)
Supplement: Supplementary file 1 — Supplementary Information [file 41467_2019_13397_MOESM1_ESM.pdf]

SUPPLEMENTARY INFORMATION

LinkedSV for detection of mosaic structural variants from linked-read exome and genome sequencing data

Fang et al.

Supplementary Figures

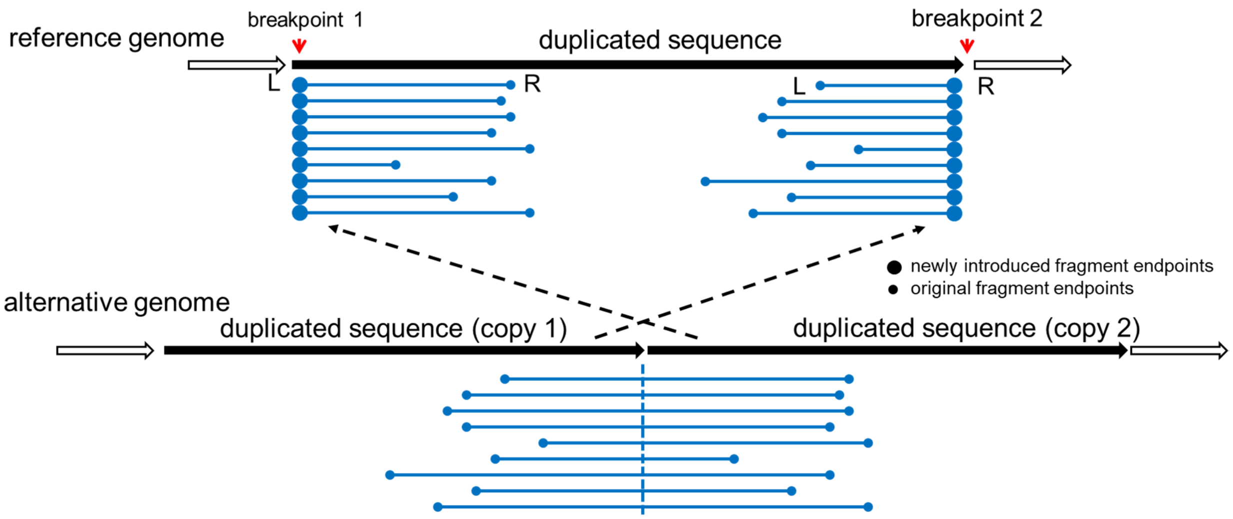

Supplementary Figure 1

The pattern of enriched fragment endpoints for tandem duplications. L-endpoints and R-endpoints are enriched near breakpoint 1 and breakpoint 2, respectively. Breakpoints are marked by red arrows.

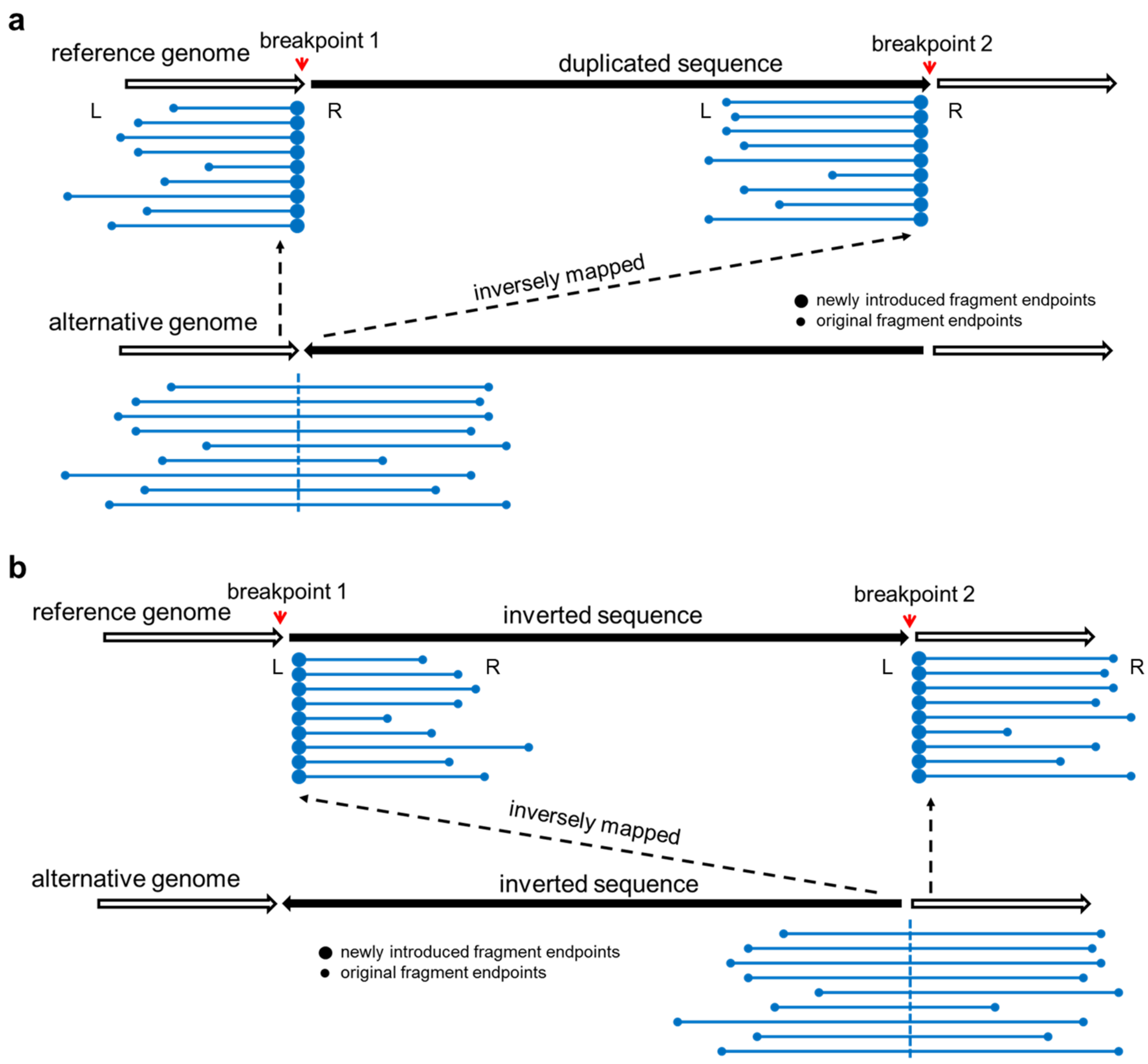

Supplementary Figure 2

The pattern of enriched fragment endpoints for inversions. The endpoints enriched near the two breakpoints are of the same type. **a**) Enriched fragment endpoints are both R-endpoints. **b**) Enriched fragment endpoints are both L-endpoints.

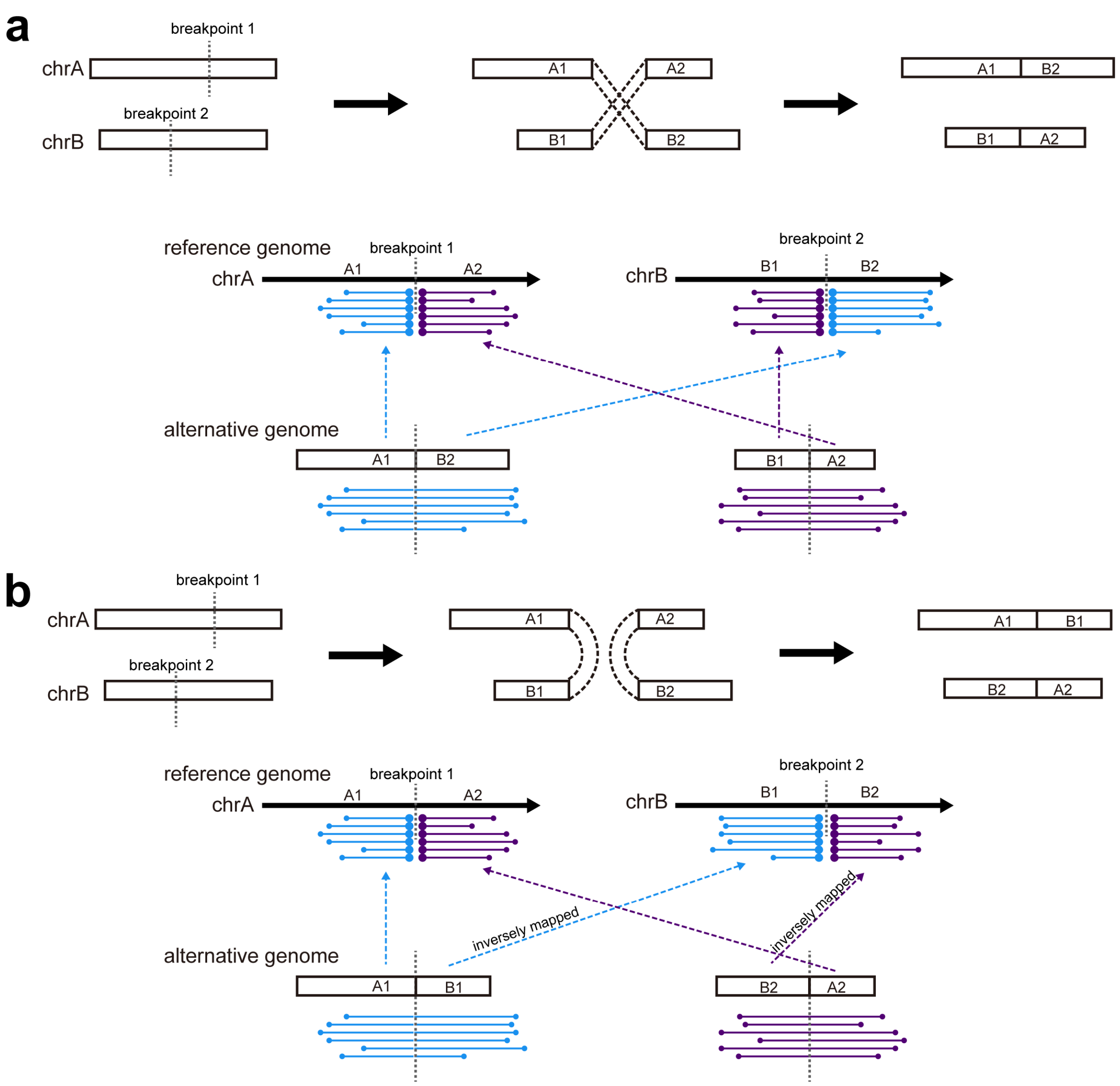

Supplementary Figure 3

The pattern of enriched fragment endpoints for interchromosomal translocations. The enriched endpoints can be either L-endpoints or R-endpoints, depending on how the two chromosomes are joined. **a**) two chromosomes are joined in the same direction. **b**) two chromosomes are joined in the reverse direction.

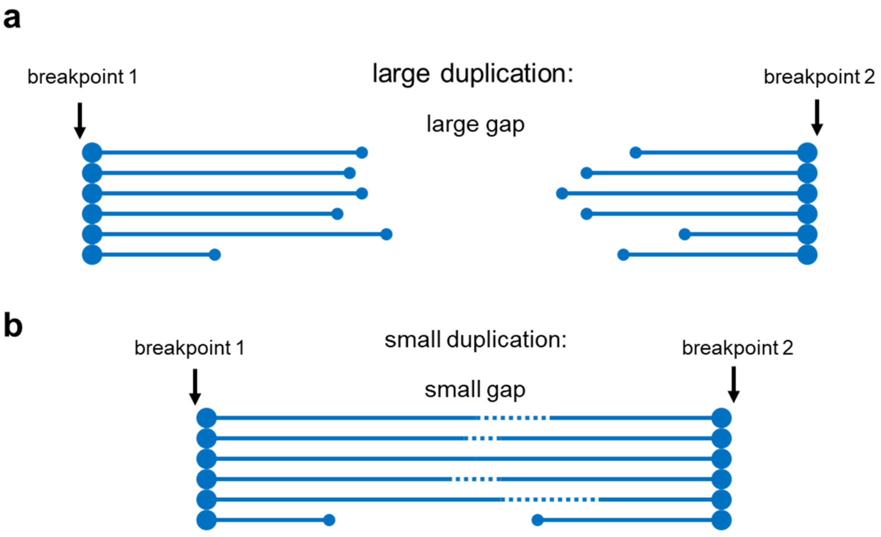

Supplementary Figure 4

a) For large duplications, the reads of the alternative allele are separated by a large gap so that we can observe two sets of fragments with the same set of barcodes, which indicate an SV. **b**) If the duplication is not large enough, the reads will be probably clustered into one fragment.

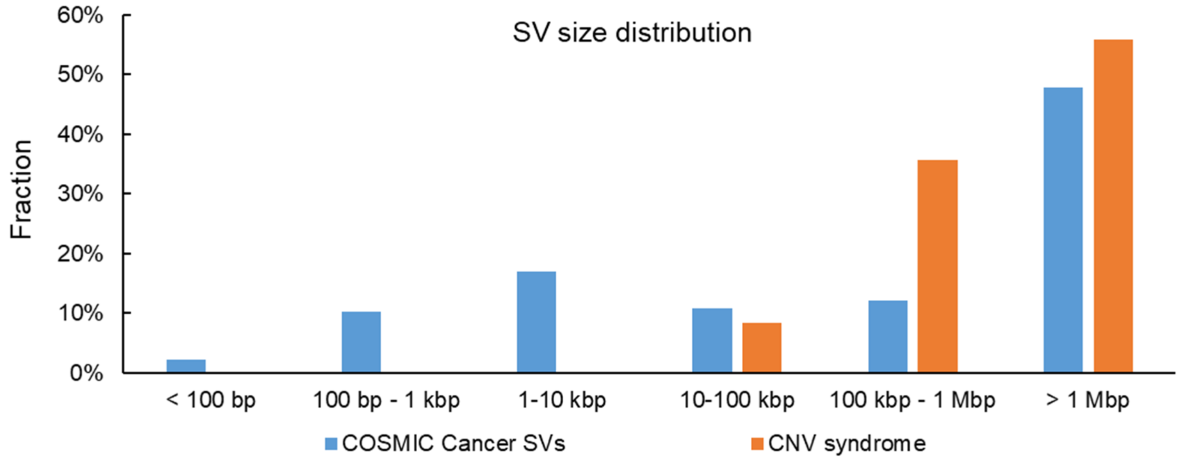

Supplementary Figure 5

Size distribution of SVs from two resources: cancer somatic SVs in COSMIC database and expert-curated known CNVs that cause CNV syndromes. Inter-chromosomal events are regarded as > 1Mbp here because in terms of SV detection using linked-reads, the inter-chromosomal events share the same properties with super large intra-chromosomal events. Source data is provided as a Source Data file.

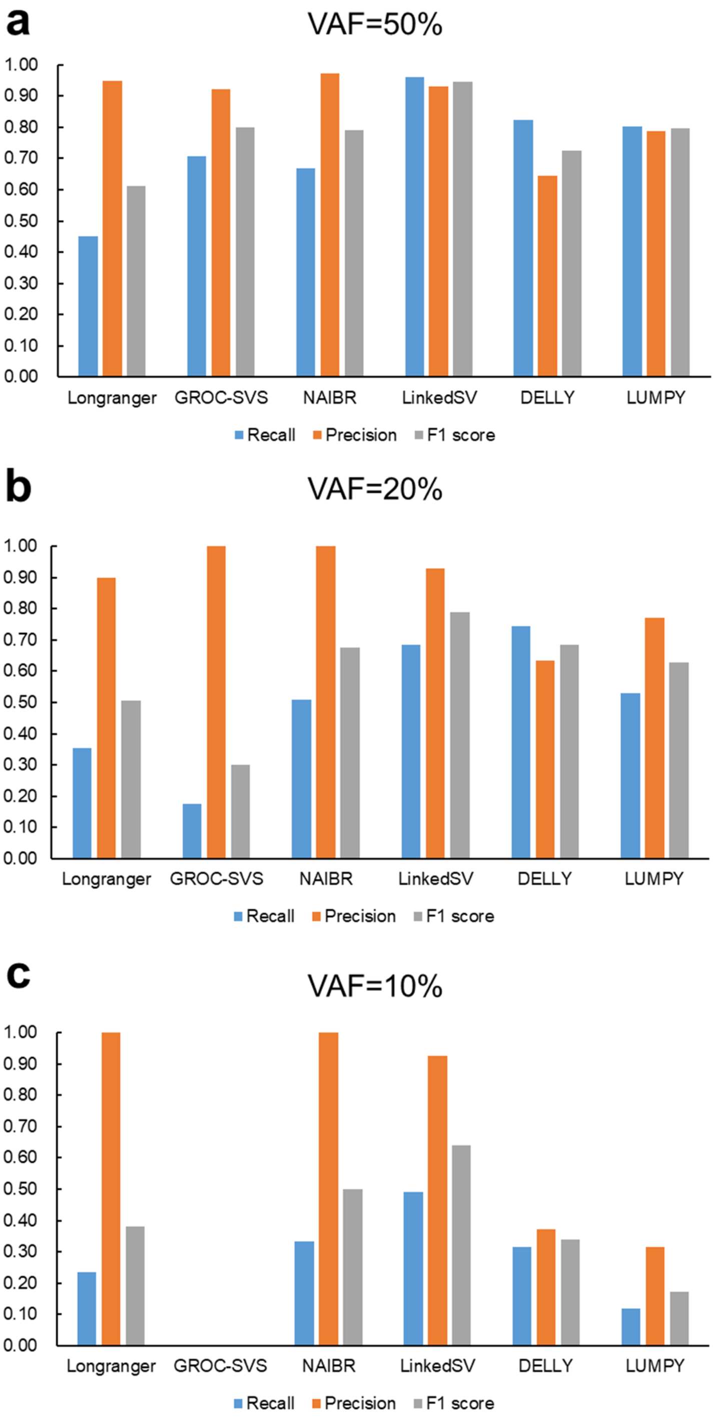

Supplementary Figure 6

Benchmarking of six SV callers on simulated deletions and duplications that cause CNV syndromes. **a**) Variant allele frequency (VAF) = 50%. This is a simulation of germline variants. **b**)

VAF = 20%. **c**) VAF = 10%. **b**) and **c**) are the simulations of somatic or mosaic variants. Source data is provided as a Source Data file.

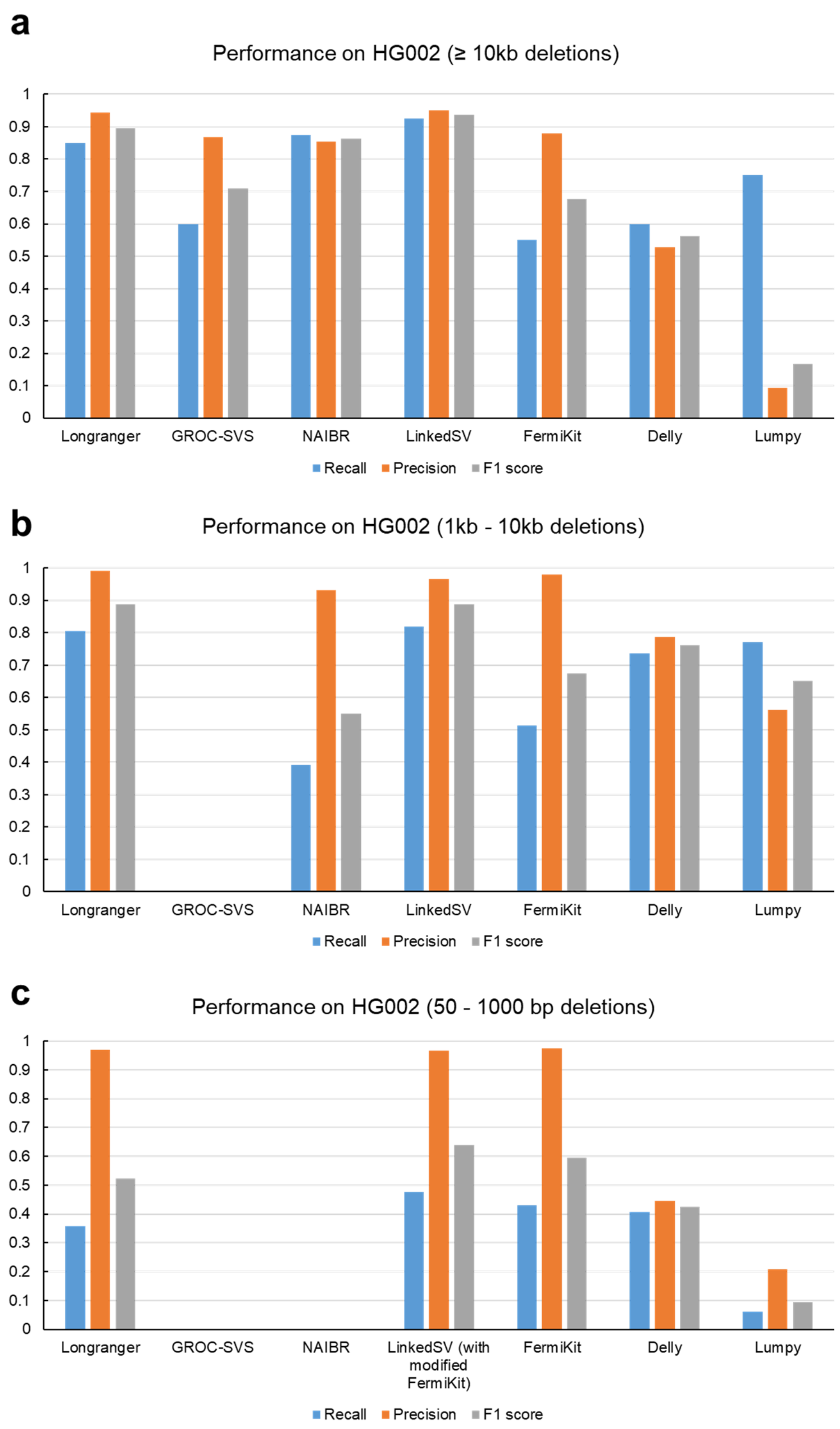

Supplementary Figure 7

Benchmarking of deletion detection on the HG002 genome. **a**) Performance of detection of deletions that are > 10 kb. **b**) Performance of detection of deletions that are within 1-10 kb. **c**) Performance of detection of deletions that are within 50-1000 bp. Source data is provided as a Source Data file.

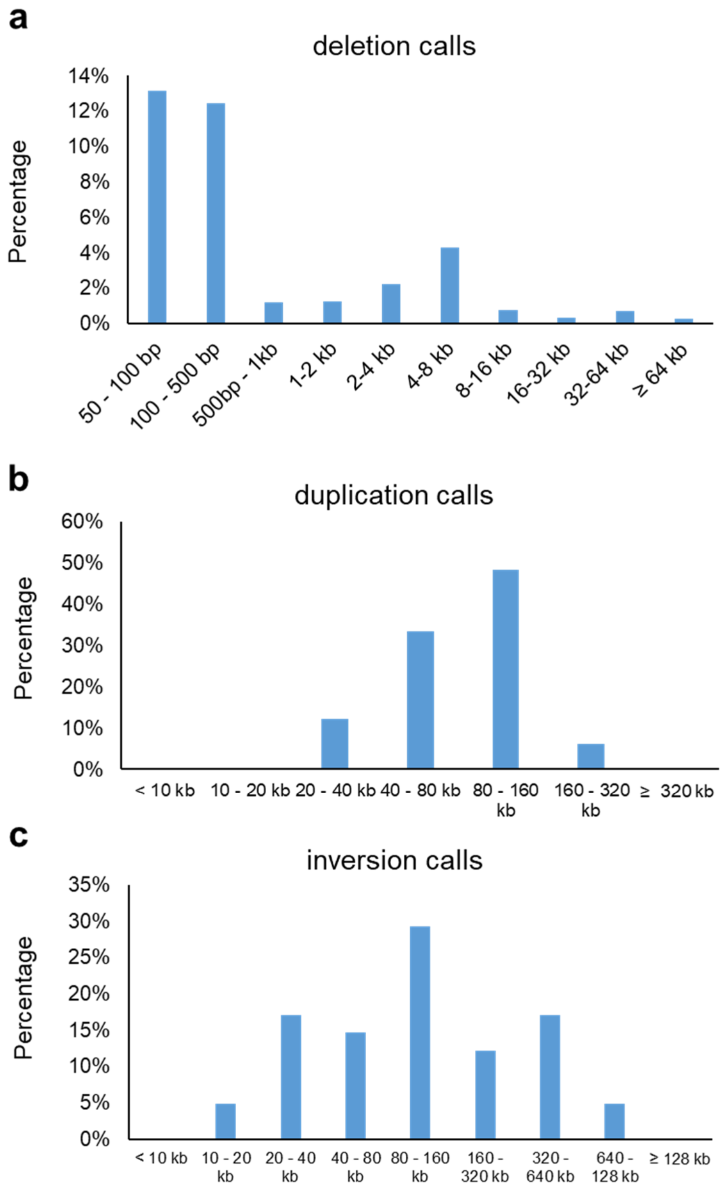

Supplementary Figure 8

Size distribution of SV events detected from the HG002 genome using LinkedSV. **a**) Deletions. The small peak in 4-8 kb indicate the events of LINE elements, which are about 7 kb long. **b**)

Duplications. **c**) Inversions. LinkedSV is able to detect small deletions up to 50 bp by using multiple information including paired-end read signals and local assembly (using modified FermiKit). LinkedSV currently only uses barcode information to detect duplications and inversions, therefore, only large duplications and inversion can be detected. Source data is provided as a Source Data file.

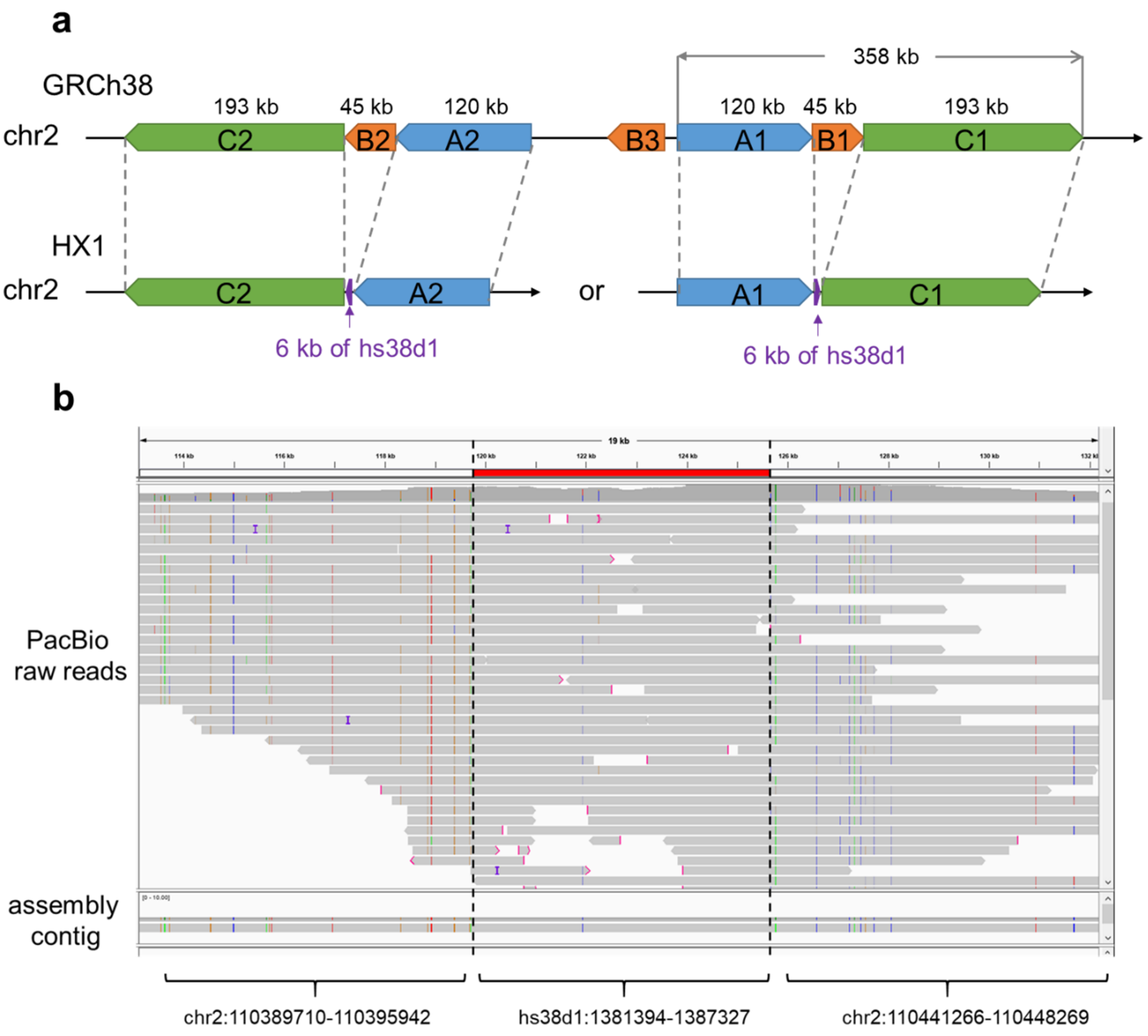

Supplementary Figure 9

a) Proposed variant allele in HX1 chr2. The 45 kb deletion region resides in a 358 kb segmental duplication region, which has two copies in chr2. The two copies are in the opposite direction and

are highly identical (fraction of matched bases = 99.88%, according to UCSC genome browser). The 45 kb deletion region has a third copy between the two 358 kb segmental duplications. In the HX1 genome, the 45 kb region is deleted and a 6 kb region from hs38d1 decoy sequence is inserted. Since the two 358 kb segmental duplications are large and highly identical, with the current read length and sequencing error rate, we are unable to tell which copy contains this event. **b**) Alignment of PacBio raw reads as well as the assembly of the aligned reads to the proposed variant allele sequence (A1 + 6 kb insertion + C1). Reads with mapping quality > 20 were shown.

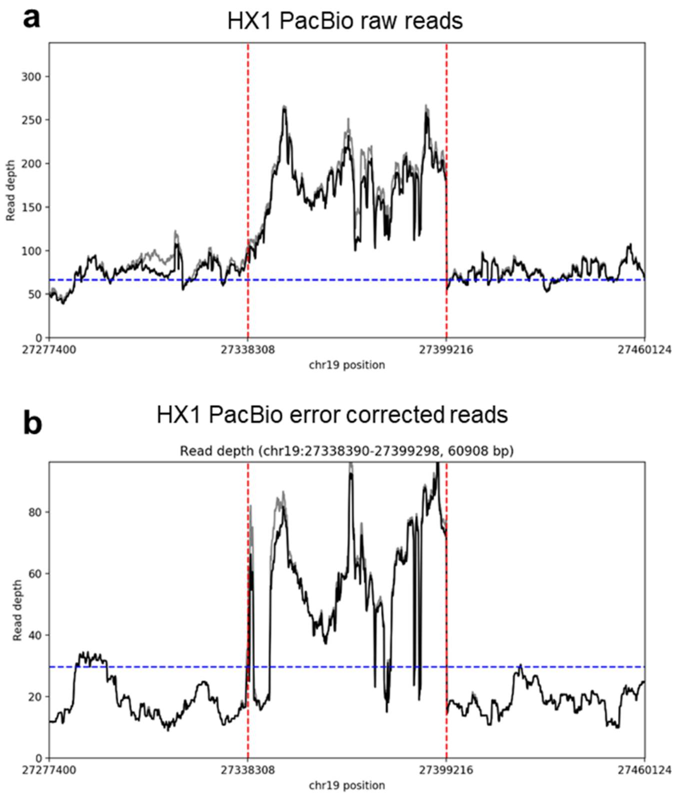

Supplementary Figure 10

Sequencing coverage of HX1 raw reads (**a**) and error corrected reads (**b**) near the chr19 duplication region detected by LinkedSV. Sequencing coverage of the linked reads is shown in Figure 7d. The predicted breakpoints by LinkedSV were indicated by vertical red lines. The dotted blue line showed the average depth across the whole genome.

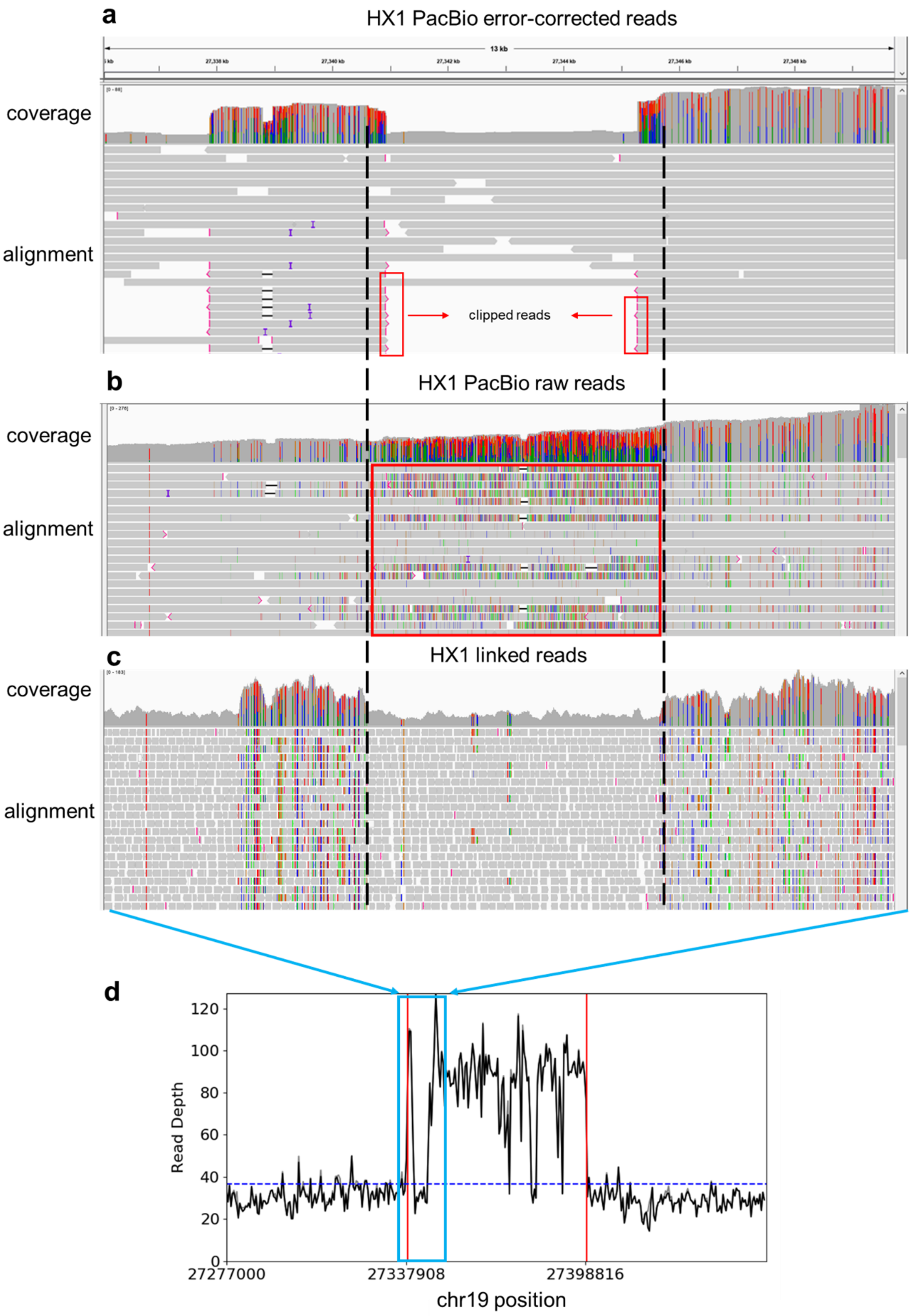
a
HX1 PacBio error-corrected reads
13 kb
coverage
alignment
clipped reads
b
HX1 PacBio raw reads
coverage
alignment
c
HX1 linked reads
coverage
alignment
d
Read Depth
0
20
40
60
80
100
120
27277000
27337908
27398816
chr19 position

Supplementary Figure 11

Inspection of the start location of the chr19 duplication in HX1 rewiews two duplication events. **a**) Sequencing coverage and alignments of error-corrected PacBio reads, as shown by IGV. A small duplication event was found next to the main event. From the coverage track, clear boundaries of the two events can be seen. In the alignment track, the clipped reads were marked by pink lines (5'-clipping) or pink arrows (3'-clipping). The alignments were genrated by minimap2 with parameters for PacBio read (-x map-pb) **b**) Sequencing coverage and alignments of raw PacBio reads, as shown by IGV. In the alignment track, mismatch bases were shown in colors (A, green; T, red; C, blue; G, orange). There are enriched alignment mismatch in the red box, indicating that this portion of reads should be clipped, rather than aligned. This may explain that the boundaris of the two events were not clear in the the coverage track. The alignments were genrated by minimap2 with parameters for PacBio read (-x map-pb). **c**) Sequencing coverage and alignments of 10X Genomics linked reads, as shown by IGV. In the coverage track, the boundaries of the two events can be seen and they are consitent with error-corrected PacBio reads. **d**) Zoom out view of the whole duplication region. Y-axis shows the sequencing coverage of 10X Genomics linked reads. The dotted blue line showed the average depth across the whole genome. The predicted breakpoints by LinkedSV were indicated by vertical red lines. This pannel is the same as Figure 7d.

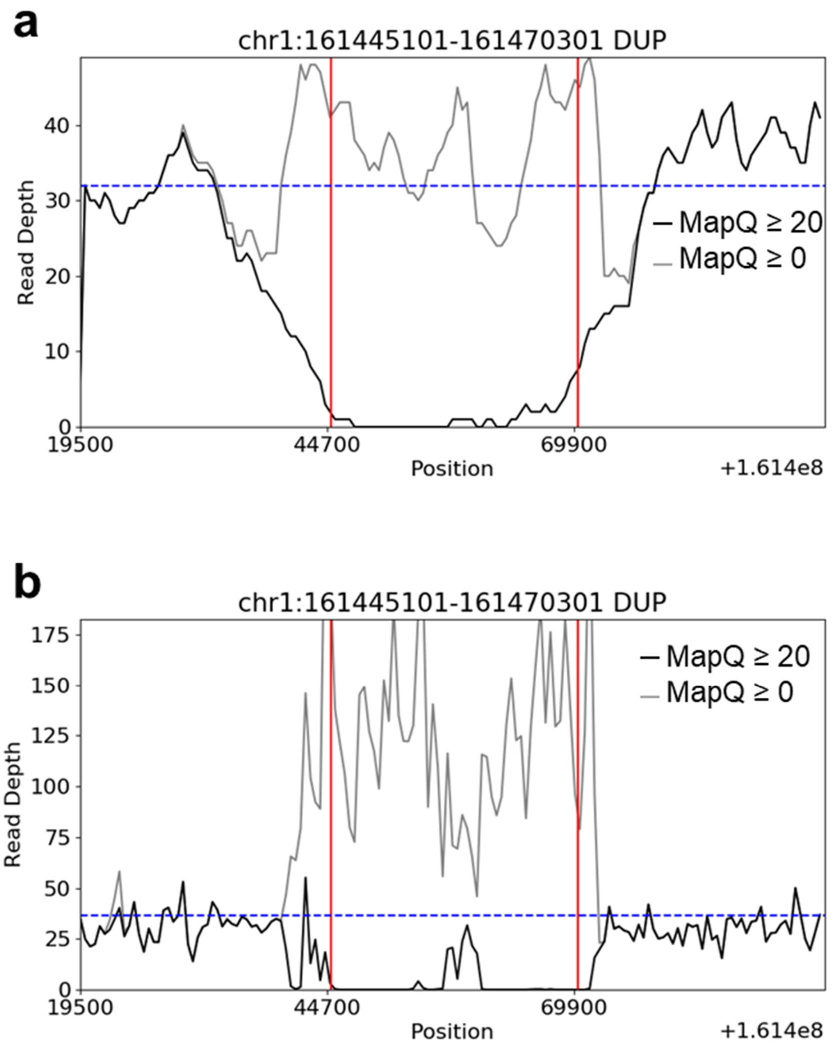

Supplementary Figure 12

Read depth distribution of PacBio long reads (**a**) and linked reads (**b**) near a duplication reported by SMRT-SV. The black lines showed the read depth of reads with mapping quality ≥ 20 while the grey lines showed the read depth of reads with mapping quality ≥ 0 (i.e. all reads). The read depth was calculated using SAMtools. The dotted blue line showed the average depth across the whole genome. The large space between the black line and the grey line indicates poor mapping qualities in the predicted duplication region.

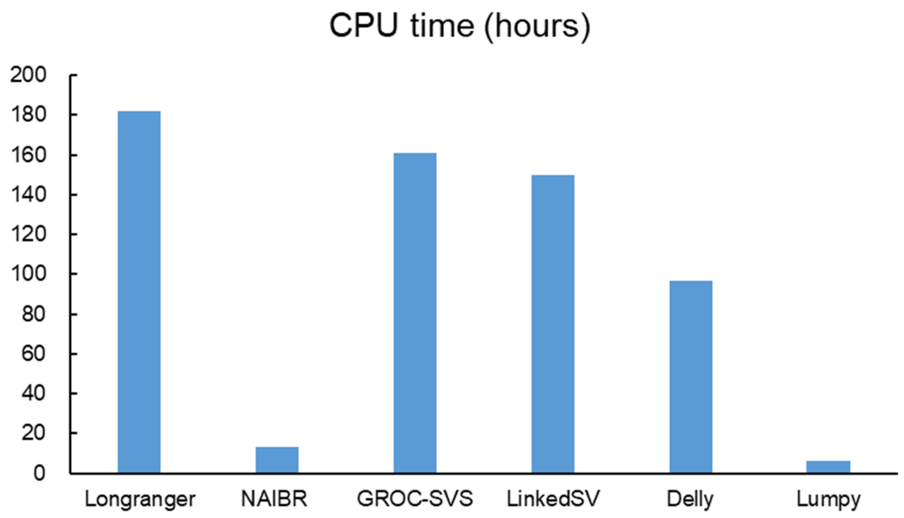

Supplementary Figure 13

Computation time (in hours) of different SV callers on the 37X coverage HX1 WGS data set. LinkedSV uses longer time than NAIBR, Delly and Lumpy, because it uses two types of barcode evidence and also performed local assembly to detect small deletions. Source data is provided as a Source Data file.

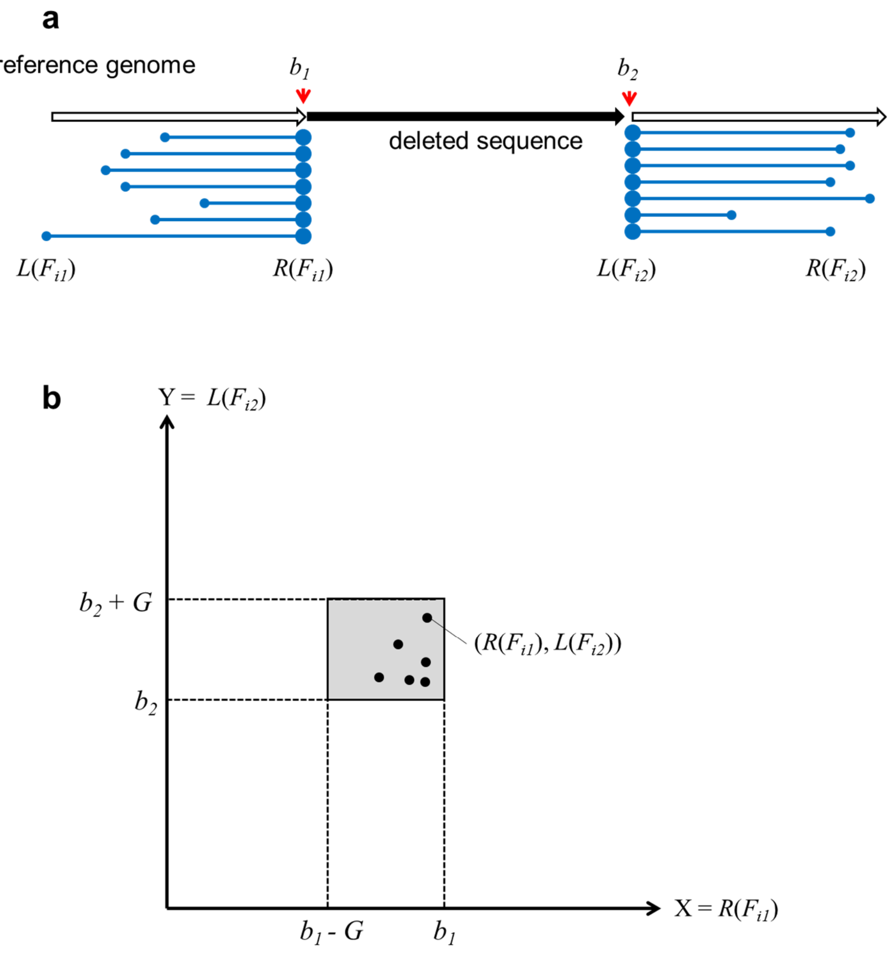

Supplementary Figure 14

Detection of type 1 evidence. We use deletion as an example but the method is also suitable for the other SV types. **a**) R-endpoints ($R(F_{i1})$) and L-endpoints ($L(F_{i2})$) are enriched near the deletion breakpoints b_1 and b_2, respectively. **b**) Two-dimensional plot of $R(F_{i1})$ and $L(F_{i2})$. $R(F_{i1})$ and $L(F_{i2})$ are restricted in the grey square according to equation (2). The background noise of the two-dimensional plot is cleaner than the one-dimensional plot since the fragments that do not share barcodes are excluded.

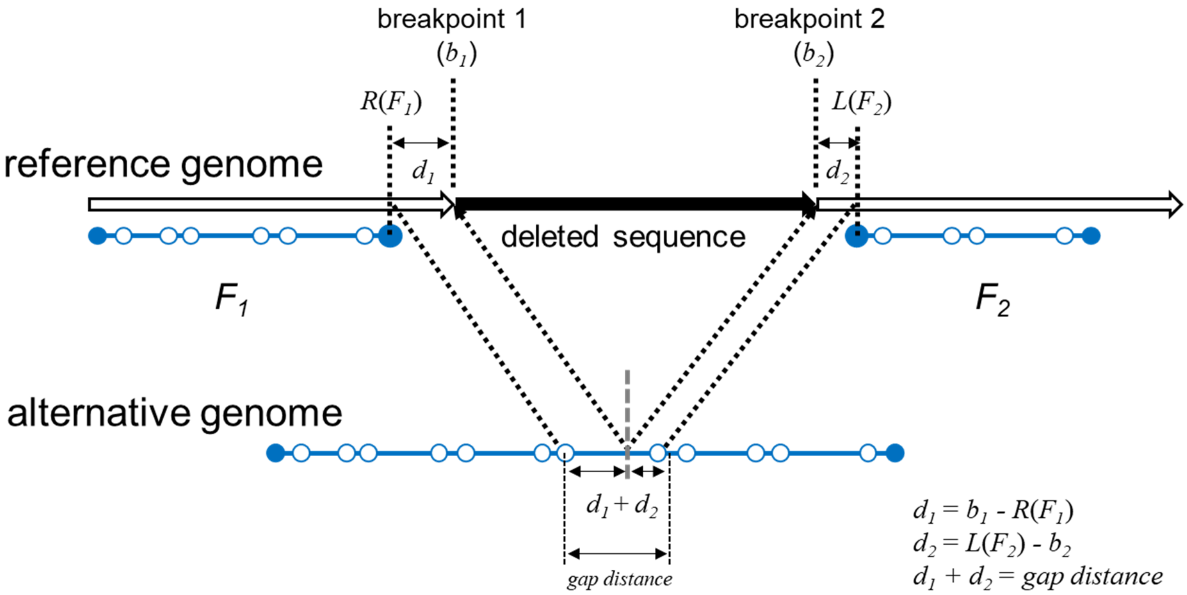

Supplementary Figure 15

Explanation of the enrichment of fragment endpoints near the breakpoints, using a deletion as an example. In this event, reads from a HMW DNA molecule that spans the breakpoints of a deletion were mapped to two genomic locations, resulting in two observed fragments (denoted by F_1 and F_2). b_1 and b_2 denote the positions of breakpoint 1 and 2. $R(F_1)$ denotes the right endpoint of F_1 and $L(F_2)$ denotes the left endpoint of F_2. d_1 and d_2 denote the distances between the fragment endpoints ($R(F_1)$, $L(F_2)$) and the corresponding breakpoints (b_1, b_2), respectively. In the original fragment of the alternative genome, $d_1 + d_2$ equals to the distance between two adjacent reads (i.e. gap distance). Therefore, gap distance is the upper limit of d_1 and d_2. Solid blue dots are endpoints of the fragments. Hollow circles are the short reads in the fragments.

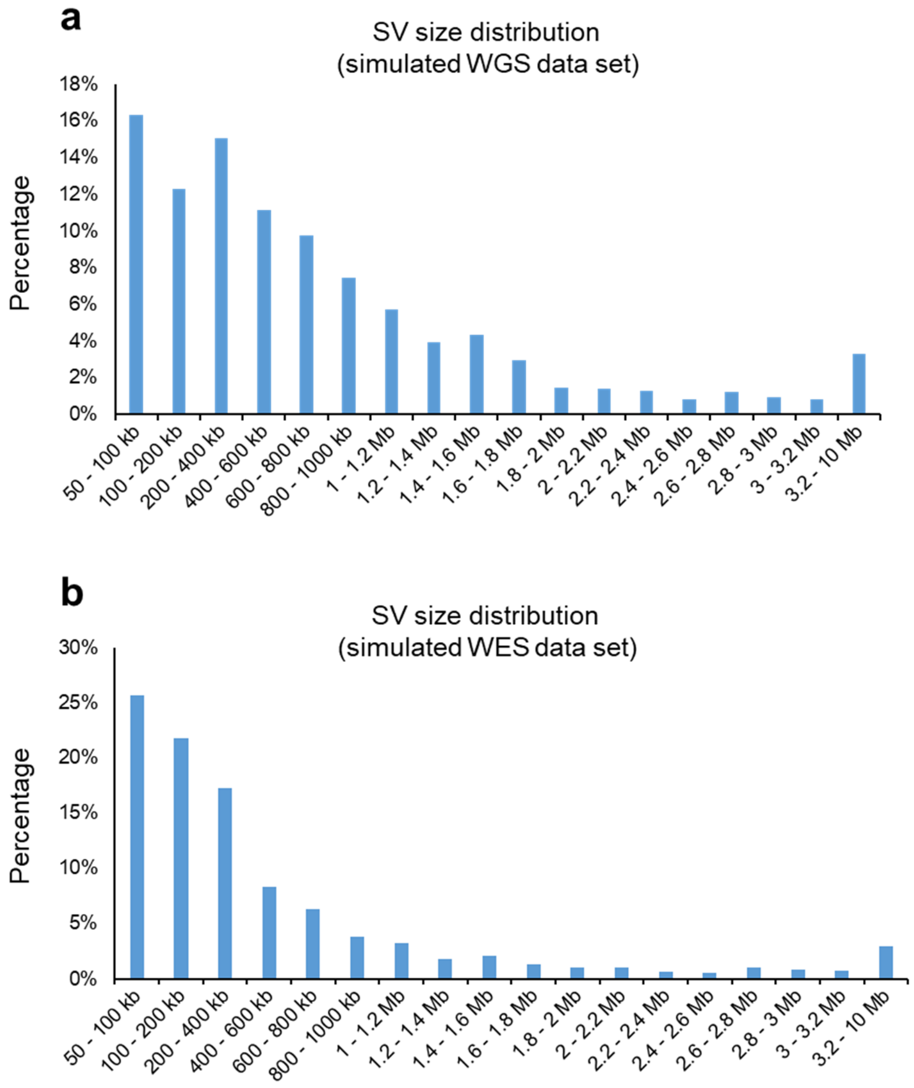

Supplementary Figure 16

Size distribution of simulated SV events. **a**) Size distribution of the SVs in the simulated WGS data set. **b**) Size distribution of the SVs in the simulated WES data set. Source data is provided as a Source Data file.

Supplementary Tables

Supplementary Table 1

SV calls detected by Delly on the *F8* inversion sample (10 kb upstream/downstream of the inversion).

Chrom	Position 1	Position 2	SV Type
chrX	154002063	154002446	INV
chrX	154004508	154004709	INV
chrX	154005670	154005855	INV
chrX	154011709	154012062	DUP
chrX	154012155	154012297	INV
chrX	154014432	154014542	INV
chrX	154014535	154014759	INV
chrX	154017795	154017948	INV
chrX	154020109	154020407	INV
chrX	154045287	154045525	INV
chrX	154045633	154045781	INV
chrX	154057649	154057833	INV
chrX	154057785	154057923	INV
chrX	154083952	154084185	INV
chrX	154091092	154091440	INV
chrX	154115196	154115584	INV
chrX	154115667	154115957	INV
chrX	154124101	154124495	DUP
chrX	154129769	154129919	INV
chrX	154130086	154130222	INV
chrX	154132781	154133165	DUP
chrX	154146261	154146462	INV
chrX	154146565	154146775	INV
chrX	154152621	154152772	INV
chrX	154157646	154157941	DUP
chrX	154158133	154158450	DUP
chrX	154158358	154158630	INV
chrX	154159826	154160143	INV
chrX	154175838	154176055	INV
chrX	154176073	154176451	INV

chrX	154194502	154194721	INV
chrX	154208905	154209847	DUP
chrX	154209557	154209679	INV
chrX	154212475	154212843	INV
chrX	154212596	154212733	INV
chrX	154227431	154227667	INV
chrX	154272329	154272476	INV
chrX	154272335	154272592	DUP
chrX	154275546	154275664	INV
chrX	154275671	154275934	INV
chrX	154290114	154290323	INV
chrX	154344170	154344430	INV
chrX	154387813	154429972	INV
chrX	154387928	154429779	INV
chrX	154391126	154391291	INV
chrX	154426121	154426264	INV
chrX	154428351	154428890	DUP
chrX	154464614	154464716	INV
chrX	154508570	154508865	INV
chrX	154540689	154540872	INV
chrX	154609922	154610441	DUP
chrX	154610132	154610342	INV
chrX	154611498	154611783	INV
chrX	154611510	154688681	DEL
chrX	154612459	154612925	DUP
chrX	154613017	154613356	INV
chrX	154652935	154653319	DUP
chrX	154687101	154687535	DUP
chrX	154687312	154687480	INV
chrX	154688521	154688851	INV
chrX	154688714	154688969	INV
chrX	154689667	154689984	INV
chrX	154735308	154735475	INV
chrX	154755098	154755225	INV

Supplementary Table 2

SV calls detected by Lumpy on the *F8* inversion sample (all SV calls in chrX).

Chrom	Position 1	Position 2	SV Type
chrX	1413852	1414637	DEL
chrX	17520630	17520931	DEL
chrX	28749622	28749739	DUP
chrX	31189715	31189837	DUP
chrX	38145097	38145387	DUP
chrX	38145261	38145866	DUP
chrX	38145731	38145851	DEL
chrX	38145513	38145876	DEL
chrX	38145369	38145943	DUP
chrX	38145283	38145943	DUP
chrX	38145180	38145975	DUP
chrX	38145091	38145976	DUP
chrX	38145835	38145920	DEL
chrX	38145729	38145981	DUP
chrX	38145874	38145914	DEL
chrX	38145321	38145988	DUP
chrX	38145471	38145995	DUP
chrX	38145929	38146016	DUP
chrX	38145971	38146003	DEL
chrX	38145885	38146041	DUP
chrX	38145642	38146055	DUP
chrX	38146077	38146257	DUP
chrX	39932139	39932332	DUP
chrX	41788764	41788966	DUP
chrX	47308661	47308783	DUP
chrX	63411862	63412017	DUP
chrX	64063531	64063690	DUP
chrX	76890054	76890207	DUP
chrX	101912168	101912314	DUP
chrX	142794887	142795158	DUP
chrX	149745015	149745169	DUP

Supplementary Table 3

SVs on chrX detected by Longranger on the *F8* inversion sample.

Chrom 1	Position 1	Chrom 2	Position 2	SV Type	Distance to int22h-1	Distance to int22h-3
chrX	3735199	chrX	3855112	Unknown	-150380286	-150832314
chrX	7810783	chrX	8151613	Unknown	-146304702	-146535813
chrX	7810783	chrX	8113116	Unknown	-146304702	-146574310
chrX	134855892	chrX	134985727	Unknown	-19259593	-19701699
chrX	152225317	chrX	152352113	Unknown	-1890168	-2335313
chrX	154091033	chrX	154660067	Unknown	-24452	-27359
chrX	154131339	chrX	154735755	Unknown	15854	48329

Supplementary Table 4

SVs detected by GROC-SVs on the *F8* inversion sample.

Chrom 1	Position 1	Chrom 2	Position 2	Orientation
chr1	12889258	chr1	12938883	+-
chr1	12919205	chr1	12853022	--
chr1	21754487	chr1	21794667	+-
chr1	148026036	chr1	144622101	++
chr1	223725671	chr1	223797843	+-
chr2	98162357	chr2	97860318	--
chr2	149687145	chr2	149790581	+-
chr2	234053740	chr2	234002965	-+
chr3	129809651	chr3	129762840	-+
chr4	9452594	chr4	9485059	+-
chr4	69681327	chr4	69893173	--
chr5	155137378	chr5	155188725	+-
chr5	180430554	chr5	180375038	-+
chr6	29909733	chr6	29843849	--
chr6	29913575	chr6	29844437	++
chr6	160956444	chr6	160877743	--
chr7	100550750	chr7	100609409	--
chr7	100555813	chr7	100610572	++
chr11	1162747	chr11	1212758	+-
chr11	5809264	chr11	5777102	-+
chr12	7239875	chr12	7189849	-+
chr12	11545335	chr12	11503243	-+
chr12	18018173	chr12	17922871	--
chr12	109372790	chr12	109423610	+-
chr12	132926655	chr16	86452753	+-
chr12	133041064	chr2	231869678	+-
chr13	114325993	chr13	114425990	+-
chr14	24436900	chr14	24474868	+-
chr15	20740860	chr15	23406226	+-
chr15	22743596	chr15	23572115	+-
chr15	23407964	chr15	20739466	+-
chr15	23573198	chr15	22742499	+-
chr15	28597075	chr15	28806596	++
chr15	28804860	chr15	28595402	--
chr15	83003956	chr15	82934463	--

chr15	83014625	chr15	82936703	++
chr15	84960511	chr15	84859997	++
chr16	14988609	chr16	15031370	--
chr16	70009791	chr16	74426101	-+
chr16	86453332	chr12	132926283	+-
chr17	36297237	chr17	36337601	+-
chr20	1600172	chr20	1559471	-+
chr20	56771729	chr12	2828954	--
chr20	56771963	chr12	2830313	++
chr22	18666166	chr22	18737108	--
chr22	18737933	chr22	18686213	++

Supplementary Table 5

SVs on chrX detected by NAIBR on the *F8* inversion sample.

Chrom 1	Position 1	Chrom 2	Position 2	Orientation	Distance to int22h-1	Distance to int22h-3
chrX	1399792	chrX	1400312	+-	-152715693	-153287114
chrX	26179787	chrX	26212951	++	-127935698	-128474475
chrX	49162006	chrX	49180528	++	-104953479	-105506898
chrX	49208629	chrX	49209218	+-	-104906856	-105478208
chrX	49218203	chrX	49218751	+-	-104897282	-105468675
chrX	52830446	chrX	52830826	+-	-101285039	-101856600
chrX	57147470	chrX	57162963	++	-96968015	-97524463
chrX	129651865	chrX	129652250	+-	-24463620	-25035176
chrX	140140043	chrX	140140427	+-	-13975442	-14546999
chrX	154387911	chrX	154430070	--	272426	-257356
chrX	155245001	chrX	155245507	+-	1129516	558081
chrX	155251108	chrX	155252593	+-	1135623	565167

Supplementary Table 6

SVs on chr17 detected by NAIBR on the *NF1* sample.

Chrom 1	Position 1	Chrom 2	Position 2	Orientation	Distance to breakpoint 1	Distance to breakpoint 2
chr17	10359246	chr17	10409257	--	-19325000	-19413270
chr17	18296487	chr17	18296856	+-	-11387759	-11525671
chr17	18314761	chr17	18315123	+-	-11369485	-11507404
chr17	18327938	chr17	18328330	+-	-11356308	-11494197
chr17	18343774	chr17	18344042	+-	-11340472	-11478485
chr17	18390522	chr17	18391011	+-	-11293724	-11431516
chr17	27963239	chr17	27964080	+-	-1721007	-1858447
chr17	34502325	chr17	34502718	+-	4818079	4680191
chr17	39241002	chr17	39296331	-+	9556756	9473804
chr17	39241014	chr17	39274415	--	9556768	9451888
chr17	39254370	chr17	39279917	-+	9570124	9457390
chr17	39382979	chr17	39411518	-+	9698733	9588991
chr17	39421637	chr17	39427775	+-	9737391	9605248
chr17	39421677	chr17	39432260	+-	9737431	9609733
chr17	39502590	chr17	39521159	--	9818344	9698632
chr17	40653142	chr17	40697232	++	10968896	10874705
chr17	41008335	chr17	41026393	++	11324089	11203866
chr17	43616656	chr17	43616962	+-	13932410	13794435
chr17	58073089	chr17	58073319	+-	28388843	28250792
chr17	58078460	chr17	58078688	+-	28394214	28256161
chr17	60343141	chr17	60343406	+-	30658895	30520879
chr17	60359848	chr17	60360239	+-	30675602	30537712
chr17	62213073	chr17	62214273	+-	32528827	32391746
chr17	62885755	chr17	62886258	+-	33201509	33063731
chr17	64794832	chr17	64795449	+-	35110586	34972922
chr17	78287778	chr17	78289508	+-	48603532	48466981
chr17	80105167	chr17	80106015	+-	50420921	50283488

Supplementary Table 7

SV calls detected by Delly on the *NF1* deletion sample (1 Mb upstream/downstream of the deletion).

Chrom	Position 1	Position 1	SV Type	SV Length
chr17	17363396	79498375	INV	62134980
chr17	17363532	64795530	INV	47431999
chr17	17363628	79499683	INV	62136056
chr17	18286739	74017685	INV	55730947
chr17	18286866	74017502	INV	55730637
chr17	28749720	28749884	INV	165
chr17	28749894	28750067	INV	174
chr17	28778475	28778674	INV	200
chr17	28778804	28778982	INV	179
chr17	28781391	28781646	INV	256
chr17	28789399	28789573	INV	175
chr17	28804180	28804452	INV	273
chr17	28850972	28851083	INV	112
chr17	28880548	28880795	INV	248
chr17	28883413	28883543	INV	131
chr17	28883874	28884185	DUP	312
chr17	28885891	28885977	INV	87
chr17	28886217	28886887	DUP	671
chr17	28886790	28887002	INV	213
chr17	28896496	28896657	INV	162
chr17	28898325	28898507	INV	183
chr17	28898453	28898776	INV	324
chr17	28900688	28900885	INV	198
chr17	28958756	28958860	INV	105
chr17	28963620	28963782	INV	163
chr17	29161159	29161443	INV	285
chr17	29214324	29214454	INV	131
chr17	29233308	29233456	INV	149
chr17	29235695	29235926	INV	232
chr17	29272077	29272279	INV	203
chr17	29280208	29280325	INV	118
chr17	29280249	29280542	DUP	294
chr17	29292652	29292783	INV	132

chr17	29297874	29298071	INV	198
chr17	29311663	29311773	INV	111
chr17	29312096	29312227	INV	132
chr17	29323985	29324271	INV	287
chr17	29328060	29328325	INV	266
chr17	29374344	29374472	INV	129
chr17	29375112	29375224	INV	113
chr17	29375965	29376167	INV	203
chr17	29376169	29376747	DUP	579
chr17	29376450	29376678	INV	229
chr17	29376869	29377109	INV	241
chr17	29377045	29377508	DUP	464
chr17	29377115	29377278	INV	164
chr17	29422253	29422388	INV	136
chr17	29442254	29442686	DUP	433
chr17	29442303	29442458	INV	156
chr17	29482195	29482490	INV	296
chr17	29496377	29496593	INV	217
chr17	29499399	29499499	INV	101
chr17	29499572	29499701	INV	130
chr17	29509791	29509902	INV	112
chr17	29553393	29553609	INV	217
chr17	29559956	29560152	INV	197
chr17	29631993	29632186	INV	194
chr17	29646082	29646292	INV	211
chr17	29653050	29653191	INV	142
chr17	29653320	29653427	INV	108
chr17	29670886	29671266	INV	381
chr17	29688359	29688466	INV	108
chr17	29691368	29691611	INV	244
chr17	29792500	29792713	INV	214
chr17	29844524	29845097	DUP	574
chr17	29844528	29844681	INV	154
chr17	29844742	29844865	INV	124
chr17	29848528	29848642	INV	115
chr17	29848670	29848862	INV	193
chr17	29897228	29897443	INV	216
chr17	29897946	29898219	INV	274
chr17	29898357	29898503	INV	147

chr17	30032123	30032285	INV	163
chr17	30032425	30032534	INV	110
chr17	30098240	30098400	INV	161
chr17	30185676	30185833	INV	158
chr17	30262037	30262472	DUP	436
chr17	30315050	30315340	INV	291
chr17	30321519	30321682	INV	164
chr17	30347576	30348121	INV	546
chr17	30348192	30348503	INV	312
chr17	30348913	30349157	INV	245
chr17	30362642	30362791	INV	150
chr17	30411530	30411734	INV	205
chr17	30418743	30418959	INV	217
chr17	30477548	30477908	INV	361
chr17	30537828	30538161	DUP	334
chr17	30537980	30538161	INV	182
chr17	30594987	30595297	INV	311
chr17	30601399	30601626	INV	228
chr17	30615745	30615901	INV	157
chr17	30662014	30662173	INV	160
chr17	30689981	30690196	INV	216
chr17	30692181	30692341	INV	161
chr17	30692457	30692615	INV	159
chr17	30693806	30693985	INV	180
chr17	30694745	30694950	INV	206
chr17	30771326	30771549	INV	224
chr17	30800970	30801130	INV	161
chr17	30806902	30807104	INV	203
chr17	30806952	30807125	INV	174
chr17	30822023	30822151	INV	129

Supplementary Table 8

SV calls detected by Lumpy on the *NF1* deletion sample (1 Mbp upstream/downstream of the deletion).

Chrom	Position 1	Position 1	SV Type	SV Length
chr17	28749892	28750050	INV	158
chr17	28804393	28804502	INV	109
chr17	28811314	28811822	INV	508
chr17	28883873	28884184	DUP	311
chr17	28886443	28886674	INV	231
chr17	28886217	28886894	DUP	677
chr17	28886812	28887273	INV	461
chr17	28898494	28898775	INV	281
chr17	29280248	29280541	DUP	293
chr17	29312021	29312222	INV	201
chr17	29376217	29376575	INV	358
chr17	29376197	29376860	DUP	663
chr17	29377124	29377287	INV	163
chr17	29377044	29377507	DUP	463
chr17	29422255	29422415	INV	160
chr17	29442358	29442535	INV	177
chr17	29499570	29499688	INV	118
chr17	29553392	29553609	INV	217
chr17	29559812	29559979	INV	167
chr17	29585386	29585478	INV	92
chr17	29631907	29632107	INV	200
chr17	29677233	29677432	INV	199
chr17	29691366	29691580	INV	214
chr17	29844728	29844864	INV	136
chr17	29844523	29844898	DUP	375
chr17	29848568	29848854	INV	286
chr17	30594805	30594919	INV	114
chr17	30594966	30595296	BND	330
chr17	30692180	30692359	INV	179
chr17	30806472	30806667	INV	195
chr17	30806578	30807234	DUP	656
chr17	18286866	74017503	BND	55730637
chr17	7167959	30145402	BND	22977443

Supplementary Table 9

SVs detected by GROC-SVs on the *NF1* WES sample (on chr17)

Chrom 1	Position 1	Chrom 2	Position 2	Orientation
chr17	397512	chr17	287512	-+
chr17	287512	chr17	397512	+-

Supplementary Table 10

Simulated CNVs that are known to cause CNV syndromes (related to Supplementary Figure 7)

Chrom	Position 1	Position 1	SV Type	SV Length
chr1	10001	12840260	DEL	12830259
chr1	145386506	145748068	DEL	361562
chr1	146533376	147883377	DUP	1350001
chr2	44410451	44589585	DEL	179134
chr2	59285696	61819816	DEL	2534120
chr2	196925121	205206940	DEL	8281819
chr2	239969863	240322644	DEL	352781
chr3	195726835	197344664	DUP	1617829
chr4	1569197	2110237	DEL	541040
chr5	10001	12533305	DEL	12523304
chr5	112043201	112181937	DEL	138736
chr5	126112314	126172713	DUP	60399
chr5	175724636	177052117	DEL	1327481
chr7	72744455	74142673	DUP	1398218
chr7	96318078	96339204	DEL	21126
chr8	8100055	11764630	DUP	3664575
chr8	77226464	77766240	DEL	539776
chr9	140513443	140730579	DEL	217136
chr11	31806339	32457088	DEL	650749
chr11	43994800	46052451	DEL	2057651
chr12	1080000	1346472	DEL	266472
chr12	65071919	68645526	DEL	3573607
chr15	22749354	28438267	DEL	5688913
chr15	30910306	32445408	DEL	1535102
chr15	74412643	75972912	DEL	1560269
chr15	99357970	102521393	DUP	3163423
chr16	60001	834373	DEL	774372

chr16	3775055	3930122	DEL	155067
chr16	14986684	16486685	DUP	1500001
chr16	21475060	29284078	DUP	7809018
chr16	29606852	30199856	DUP	593004
chr17	1	2588910	DEL	2588909
chr17	14097915	15470904	DUP	1372989
chr17	16773072	20222150	DUP	3449078
chr17	29107097	30263322	DEL	1156225
chr17	34815072	36215918	DEL	1400846
chr17	43705166	44294407	DEL	589241
chr21	27252860	27543447	DUP	290587
chr22	1	16971861	DUP	16971860
chr22	19009792	21452446	DEL	2442654
chr22	21917117	23722446	DEL	1805329
chr22	51045516	51187845	DEL	142329
chrX	751878	867876	DEL	115998
chrX	6455812	8133196	DEL	1677384
chrX	48334549	52117662	DUP	3783113
chrX	53401070	53683276	DUP	282206
chrX	103031438	103047548	DUP	16110
chrX	153287263	153363189	DUP	75926
chrX	153624563	153881854	DUP	257291
chrY	14352761	15154863	DEL	802102
chrY	20118045	26065198	DEL	5947153

Supplementary Methods

Detection of SVs using different SV callers

SV detection using Longranger (version 2.2.2) was performed with default settings for detection of germline SVs, and the "--somatic" parameter was set for detection of SVs with VAF of 10% and 20%. SV detection using GROC-SVs (version 0.2.5) was performed using default settings with a blacklist file from 10X Genomics. SV detection using NAIBR (commit 15eba96) was performed using default settings a blacklist file from 10X Genomics. SV detection using Delly (version 0.8.1) was performed with default settings. SV detection using Lumpy (version 0.3.0) was performed using the smoove pipeline (https://github.com/brentp/smoove) according to the authors' suggestions. *De novo* assembly based SV calling using FermiKit (commit bf9c711) was performed with the parameters "-s 3g -l 151" to specify the expected genome size and read length. Detection of SVs from the HX1 PacBio data set using Sniffles (version 1.0.11) was performed with the "--min_support 1" parameter to set the minimum number of reads that support an SV to be 1. The purpose is to maximize the sensitivity and see if Sniffles can detect the duplications reported by SMRT-SV.

Error correction of PacBio reads

PacBio sequencing data of the HX1 genome was downloaded from NCBI SRA database with the accession SRX1424851. Raw PacBio reads (subreads) were extracted from the HDF5 files using pbh5tools according to the manufacturer's instructions. Error correction of PacBio reads was performed using Canu[1] (version 1.6) with the parameters for PacBio reads (canu -correct genomeSize=3.1G -pacbio-raw path/to/pacbio_reads.fasta).

Mapping of PacBio data to the human genome

Raw PacBio reads or error-corrected PacBio reads were mapped to human reference genome GRCh38 (without ALT loci) using Minimap2[2] (version: 2.15-r905) with parameters optimized for PacBio reads (-ax map-pb). The output SAM file was converted to BAM file and sorted using SAMtools[3].

De novo assembly of the PacBio reads supporting the SV

SAMtools was used to extract the PacBio reads that were aligned to the proposed variant allele in HX1 (related to Supplementary Figure 9). *De novo* assembly of the extracted reads was performed using wtdbg2[4] with the parameters "-x rsII -g 50k" to specify that the platform was PacBio RS II and the estimated size of the target region was 50 kb. A single contig of 42.7 kb was generated.

Comparison of the SV call sets of LinkedSV and SMRT-SV on the HX1 genome

The SV calls of SMRT-SV were downloaded from NCBI dbVar database under accession nstd162. We only compared large SV calls that are at least 10 kb, because LinkedSV had limited power to detect smaller SVs except small deletions. The performance of small deletion detection was evaluated on the GIAB HG002 genome. Two SV calls were considered the same if they had at least 50% reciprocal overlap (the overlapped region was more than 50% of both calls). This criterion was chosen to follow what was done by a previous study [5].

Supplementary Notes

Supplementary Note 1: Explanation of the model for detecting type 2 evidence.

Barcode similarity between two nearby regions very high because the reads originate from almost the same set of HMW DNA molecules. However, the barcode similarity between the left side and right side of the breakpoint are dramatically reduced. We call this evidence as type 2 evidence. To detect type 2 evidence, LinkedSV uses two adjacent sliding windows (window 1 and window 2) to scan the genome and calculate the barcode similarity between the window 1 and window 2.

In WES data sets, the numbers of reads in the sliding windows vary a lot due to capture bias and the length of capture regions. To detect type 2 evidence from both WGS and WES data sets, our model considers the variation of sequencing depth and capture regions. The barcode similarity is calculated as:

$$S = \frac{x}{m_1^a m_2^b} n e^{-\alpha d} \quad (1),$$

where:

m_1 is the number of barcodes in window 1,

m_2 is the number of barcodes in window 2,

x is the number of barcodes in both windows,

d is the weight distance between reads of the left window and the right window,

n is a constant representing the characteristic of the library,

α is a parameter of fragment length distribution,

a and b are two parameters between 0 and 1,

n, α, a and b are estimated from the data using regression.

Suppose there are *n* different HMW DNA molecules span both window 1 and window 2, each of which has a different barcode and generates a number of read pairs in the library. The read pairs in the library may or may not be sequenced. We assume the *n* HMW DNA molecules have the same rate to generate read pairs in the library, so the *n* HMW DNA molecules have the same chance to be sequenced (have at least 1 read). Let m_1 be the number of HMW DNA molecules sequenced in window 1, m_2 be the number of HMW DNA molecules sequenced in window 2. m_1 and m_2 can be different due to the bias of target enrichment and the total length of target regions in each window. Let X be the number of HMW DNA molecules sequenced in both window 1 and window 2. X follows the hypergeometric distribution:

$$P(X = x \ |m_1, m_2, n) = \frac{C_{m_1}^{x} C_{n-m_1}^{m_2-x}}{C_n^{m_2}} \quad (2).$$

The expectation of X is:

$$E(X) = \frac{m_1 m_2}{n} \ (3).$$

However, the length of sliding windows may be as long as 40 kb and not all the *n* HMW DNA molecules are long enough to span both windows. In addition, the capture regions in window 1 and window 2 may be close to each other or far away from each other. Therefore, we need to adjust *n* to be approximately $ne^{-\alpha d}$. *d* is calculated using the following equation:

$d = w_2 - w_1$ (4),

where w_1 is the mean mapping position of all reads in window 1 and w_2 is the mean mapping position of all reads in window 2. The larger *d*, the smaller number of HMW DNA molecules can span a region of length *d*. We choose exponential distribution because the length of HMW DNA molecules follows exponential distribution and thus the number of HMW DNA molecules longer than *d* also follows exponential distribution.

m_1 also need to be adjusted because not all HMW DNA molecules being sequenced in window 1 span both windows. We adjust m_1 to be approximately m_1^a and similarly adjust m_2 to be approximately m_2^b.

After the adjustment, the expectation of X is:

$E(X) = \frac{m_1^a m_2^b}{ne^{-\alpha d}}$ (5).

We define barcode similarity as:

$S = \frac{x}{E(X)} = \frac{x}{m_1^a m_2^b} ne^{-\alpha d}$ (6),

where x is the number of shared barcodes between window 1 and 2, $E(X)$ is the expected number of shared barcodes between window 1 and 2.

Take the log of both sides equation (6), we have:

$\log(S) = \log(x) - alog(m_1) - blog(m_2) + \log(n) - \alpha d$ (7).

Assuming most regions in the genome do not have breakpoints, we can replace S with 1 and estimate a, b, n, α from the data using linear regression.

Supplementary Note 2: Range of SV size that can be detected by LinkedSV

LinkedSV is able to detect deletions ≥ 50 bp, inversions ≥ 10 kb, tandem duplications ≥ 20 kb and intra-chromosomal translocations of any size. Supplementary Figure 8 showed the size distribution of SV events detected from the HG002 genome using LinkedSV.

LinkedSV has limited power to detect small SV duplications and inversions. Fortunately, a lot of large SVs are known to be associated with human diseases. We analyzed the sizes of SVs from the following two resources:

1) Somatic Cancer SVs released by the COSMIC database [6] (version 89, released May 15th, 2019). This data set contains 351,862 SVs.

2) Expert-curated known SVs that cause CNV syndromes. The DECIPHER (Database of Chromosomal Imbalance and Phenotype in Humans Using Ensembl Resources) database[7] provides a list of expert-curated microdeletion and microduplication syndromes involved in developmental disorders. The genomic coordinates of the CNVs were obtained from https://decipher.sanger.ac.uk/disorders#syndromes/overview . This data set contains 67 SVs.

The SV size distribution of the two data sets were shown in Supplementary Figure 5. 71% of the cancer somatic SVs released by COSMIC database are inter-chromosomal events or intra-chromosomal events that are larger than 10 kb. All deletions/duplications that cause the CNV syndromes are larger than 10 kb. The results indicated that large SVs (including inter-chromosomal SVs) are associated with diseases such as cancers and CNV syndromes. Of note, we need to be aware that there is potential bias in calculating the fraction of large CNVs, as large CNVs are easier to detect and there may exist disease-associated small CNVs that have not been detected.

Supplementary References

1. Koren S, Walenz BP, Berlin K, Miller JR, Bergman NH, Phillippy AM. Canu: scalable and accurate long-read assembly via adaptive k-mer weighting and repeat separation. *Genome Res* **27**, 722-736 (2017).

2. Li H. Minimap2: pairwise alignment for nucleotide sequences. *Bioinformatics* **34**, 3094-3100 (2018).

3. Li H*, et al.* The Sequence Alignment/Map format and SAMtools. *Bioinformatics* **25**, 2078-2079 (2009).

4. Ruan J, Li H. Fast and accurate long-read assembly with wtdbg2. Preprint at https://www.biorxiv.org/content/10.1101/530972v1 (2019).

5. Pendleton M*, et al.* Assembly and diploid architecture of an individual human genome via single-molecule technologies. *Nat Methods* **12**, 780-786 (2015).

6. Tate JG*, et al.* COSMIC: the Catalogue Of Somatic Mutations In Cancer. *Nucleic Acids Res* **47**, D941-D947 (2019).

7. Firth HV*, et al.* DECIPHER: Database of Chromosomal Imbalance and Phenotype in Humans Using Ensembl Resources. *Am J Hum Genet* **84**, 524-533 (2009).
