# Supplementary material for: LinkedSV for detection of mosaic structural variants from linked-read exome and genome sequencing data
Source: Nat Commun. 2019 Dec 6;10:5585. doi: 10.1038/s41467-019-13397-7 (PMC6898185; doi:10.1038/s41467-019-13397-7)
Supplement: Supplementary file 3 — Description of Additional Supplementary Files [file 41467_2019_13397_MOESM3_ESM.pdf]

Description of Additional Supplementary Files

Supplementary Data 1.

Simulated SVs in the WGS data set

Supplementary Data 2.

Simulated SVs in the WES data set

Supplementary Movie 1.

Explanation of enriched fragment endpoints for tandem duplications. The movie shows the mapping process of the linked reads and how the fragment endpoints are enriched near the breakpoints.
